# Influence of Digital Manufacturing and Abutment Design on Full-Arch Implant Prostheses—An In Vitro Study

**DOI:** 10.3390/ma18153543

**Published:** 2025-07-29

**Authors:** Shahad Altwaijri, Hanan Alotaibi, Talal M. Alnassar, Alhanoof Aldegheishem

**Affiliations:** 1 Department of Clinical Dental Sciences, College of Dentistry, Princess Nourah bint Abdulrahman University, P.O. Box 84428, Riyadh 11671, Saudi Arabia; smaltwaijri@pnu.edu.sa; 2 Department of Prosthetic Dental Sciences, College of Dentistry, King Saud University, Riyadh 11545, Saudi Arabia; haalotaibi@ksu.edu.sa (H.A.); talnassar@ksu.edu.sa (T.M.A.)

**Keywords:** framework, digital manufacturing, 3D printing, abutment connection design, misfit, cantilever, implant-supported FDP, full arch

## Abstract

Achieving accurate fit in implant-supported prostheses is critical for avoiding mechanical complications; however, the influence of digital manufacturing techniques and abutment designs on misfit and preload remains unclear. This study evaluated the impact of different manufacturing techniques (CAD-cast and 3D printing) and abutment connection types (engaging [E], non-engaging [NE]) on the misfit and preload of implant-supported cantilevered fixed dental prostheses (ICFDPs). Misfit was measured at six points using scanning electron microscopy, and preload was assessed via eight strain gauges placed buccally and lingually on four implants. Frameworks were torqued to 35 Ncm, retorqued after 10 min, and subjected to 200,000 cycles of loading. Mean preload values ranged from 173.4 ± 79.5 Ncm (PF) to 330 ± 253.2 Ncm (3DP). Preload trends varied depending on the abutment type and manufacturing technique, with the 3DP group showing higher preload in engaging (E) abutments, whereas the CAD-cast group showed the opposite pattern. Although preload values varied numerically, these differences were not statistically significant (*p* = 0.5). In terms of misfit, significant differences were observed between groups (*p* < 0.05), except between CAD-cast E (86.4 ± 17.8 μm) and 3DP E (84.1 ± 19.2 μm). Additionally, E and NE abutments showed significant differences in misfit within both CAD-cast and 3DP groups. Overall, 3DP frameworks showed superior fit over CAD-cast. These findings suggest that 3DP may offer improved clinical outcomes in terms of implant–abutment fit.

## 1. Introduction

Dental implants are a well-established treatment modality for the rehabilitation of edentulous patients [1,2]. Although they demonstrate high success rates, both biological and technical complications may arise—particularly in long-span implant-supported fixed dental prostheses with cantilever extension (ICFDPs) [3,4,5]. One of the most notable technical complications is the loss of screw preload at the implant–abutment connection, which can lead to screw loosening. Under functional loading, these prostheses are subject to external and internal forces that increase the number of induced stresses in all components of the prosthesis, particularly at the implant–abutment interface [6], with a 5.3% incidence of loosening after one year, increasing to between 5.8% and 12.7% after 5 years [7].

A retrospective study with a mean observation period of 9.6 years reported higher rates of screw loosening in ICFDPs compared to non-cantilever prostheses [8]. Similarly, a 5-year systematic review reported a 7.9% incidence of screw loosening in ICFDPs [9]. Various factors have been shown to influence screw preload and joint stability, including the design of the implant–abutment connection and the digital manufacturing technique used [2,3,4,5,6,7,8,9,10].

Conventional casting remains a cost-effective method for fabricating dental prostheses; however, it is associated with a high incidence of human and laboratory errors [11]. Casted frameworks often exhibit porosities, casting defects, and inferior mechanical properties compared to those produced through digital manufacturing techniques [11,12]. In contrast, computer-aided design/computer-aided manufacturing (CAD/CAM) systems represent a promising alternative, offering improved material consistency, enhanced precision, and greater quality control [11,13]. Additionally, CAD/CAM workflows reduce clinical and laboratory steps, minimize operator-dependent variability, enable a high degree of customization, and simplify the overall fabrication process [14,15].

While several studies have evaluated the influence of digital manufacturing techniques on the accuracy, morphology, and misfit of implant-supported prostheses [15,16,17,18], few have investigated their effect on abutment screw preload in comparison to conventional techniques. Furthermore, the design of the implant–abutment connection, which is considered the weakest part of the ICFDPs, has rarely been examined for its role in the screw preload. This parameter is critical in resisting occlusal forces, preventing micromotion, and decreasing bacterial microleakage at the implant–prosthesis interface [19].

Abutments used in internal implant configurations are categorized as either engaging (E) or non-engaging (NE), based on whether they include an anti-rotational feature extending from the apical end of the abutment. Engaging abutments have orientation grooves or features that prevent rotational movement by locking into the implant, providing greater stability. In contrast, non-engaging abutments lack these features and allow passive fit, which can be advantageous in multi-unit prostheses to accommodate minor discrepancies in fit. In a review summarizing the mechanism and factors associated with abutment screw loosening, clinicians preferred using NE abutments for full-mouth rehabilitation with a splinted fixed superstructure, as they achieved a good fit while allowing up to a certain degree of parallelism correction [20]. This was due to the short lateral wall of non-engaging (NE) abutments, which allows for easier insertion and removal of the superstructure, especially in cases where implant parallelism is suboptimal [20,21]. Additionally, NE abutments help reduce the amount of stress transferred to the implants during the tightening process [20,21]. However, NE abutments show a 40% incidence of screw loosening [22]. This abutment design limits the height and diameter of the implant–abutment connection, leading to a weaker interface when loaded [10,20,23]. Thus, the effect of any eccentric forces is directed solely on the abutment screw, increasing the incidence of the loss of preload and screw loosening [10,20,23].

Engaging (E) abutments have a higher collar and grooves that provide an anti-rotation mechanism and increase the stability of the framework. This high collar protects the screws from the effect of lateral forces and minimizes the stress on the abutment screw [2]. E abutments show decreased screw loosening and reduced movement between the abutment and implant [2,10,19,23], whereas NE abutments exhibit significantly high screw fractures at lower cycles with minimal wear on implant analogs [24]. Moreover, NE abutments have been shown to have higher microstrains affecting the bone [25]. In a systematic review comparing different implant–abutment connection designs, screw loosening and fractures of the abutment occurred more often in NE abutments than in E abutments [26]. However, studies have shown that using prostheses with all E abutments leads to difficulties while inserting the multiple-unit splinted restorations because of the limited path of insertion and the long lateral wall height of the engaging abutment, which has an average length of 2.4 mm [21]. Incorporating both abutment designs in implant-retained prostheses is a recent alternative that should combine the easy insertion of NE abutments and the increased stability of E abutments [20]. This design provides prosthetic convenience in insertion and removal and dissipates stress away from the abutment to the implant fixture, thus decreasing the loss of preload and screw loosening [2,10,21]. However, the question remains whether digital manufacturing techniques, in combination with engaging and non-engaging abutment designs, influence the misfit and preload behavior of full-arch cantilevered implant-supported restorations.

Therefore, the aim of this study was to evaluate the influence of digital manufacturing techniques and implant–abutment connection design on the level of misfit of ICFDPs and to compare the abutment screw’s preload monitored using strain gauges of different ICFDPs with different designs subjected to cyclic loading on the extension arm. The null hypothesis is that no differences exist in the implant–abutment misfit and abutment screw preload of ICFDPs manufactured using different digital manufacturing techniques and using E and NE abutment connections in the terminal implant next to the cantilever extension.

## 2. Materials and Methods

A mandibular edentulous master model (30 × 60 × 15 cm) was constructed using a soft silicone mold (LB003B Models Molds; Zogear Dental, Shanghai, China) and clear heat-cured acrylic resin (Clear Orthodontic resin; Dentsply Sirona, Charlotte, NC, USA).

**Figure 1:**
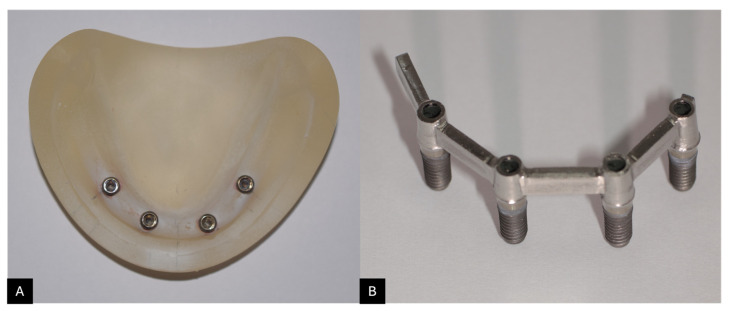
(**A**) The mandibular edentulous master model with four inter-foraminal implants. (**B**) Control framework (PF) fabricated using the CAD-cast method.

Four implant sites were prepared in the inter-foraminal region with 15 mm spacing between their centers. The implant beds were drilled first with an acrylic bur (Dentsply Sirona, NC, USA) and finalized with a 4.1 mm twist drill (Straumann, Basel, Switzerland), using a handpiece (EXPERTmatic E10C; Kavo Dental, Biberach, Germany) attached to a parallelometer (Parmax II paralleling device; WhaleDent, Arlandastad, Sweden). The distance was measured and confirmed using a digital caliper (7-511; Bernstein-Werkzeuge, Remscheid, Germany). Four 4.1 mm × 10 mm tissue-level implants (Tapered Effect; Straumann, Basel, Switzerland) were temporarily aligned parallel using a long implant driver (TE profile drill; Straumann, Basel, Switzerland) fixed to a parallelometer (Parmax II paralleling device; WhaleDent, Sweden) and stabilized with a heavy-body polyvinylsiloxane putty (Express STD Putty, 3M Espe, Seefeld, Germany) in a 1:1 catalyst-to-base ratio [27]. The implants were sequentially numbered from 1 to 4, with implant No. 1 representing the most distal position in the third quadrant (Figure 1A).

### 2.1. Control Framework Fabrication (PF Group)

A CAD-cast hybrid method was used to fabricate the control framework. Scan bodies (048.068; Straumann, Basel, Switzerland) were attached to the implants in the master model and then scanned using a digital scanner (CERamill Map 300; Amann Girrbach GmbH, Pforzheim, Germany). The obtained STL file was used to design a CAD framework, measuring 5 mm in height and 3 mm in width, featuring four non-engaging abutments (NE) and a 10-mm cantilever near implant No. 4, using Ceramill Mind software (version 3.x; Amann Girrbach GmbH, Pforzheim, Germany).

The CAD-designed framework was milled from Dima Mill wax (Kulzer GmbH, Hanau, Germany) using a Ceramill Motion 2 milling machine (Amann Girrbach, GmbH, Pforzheim, Germany). After 24 h of storage, the wax-up was embedded in a phosphate-based investment material with a water-soaked paper for one minute (K&B investment; YETI Dental, Engen, Germany). Thermal cycling was performed in a centrifugal casting machine (Centrifico; KaVo Dental, Biberach, Germany), gradually heating to 650–700° over one hour and holding for 15 min. Afterward, the investment was burned out in a furnace. To remove surface oxides, the castings were quenched, pickled, and cast into a single-piece cobalt–chromium framework (Co-Cr) alloy (StarLoy C; DeguDent GmbH, Hanau, Germany). To achieve a passive fit, the implants were detached from the master model, reassembled, and secured to the framework with connecting screws (048.350 occlusal screw; Straumann, Basel, Switzerland). The implants were fixed to the framework and bonded to the master model with extra-clear acrylic resin (Clear Orthodontic Resin; Dentsply Sirona, NC, USA). This framework was used as a control and was referred to as PF (Figure 1B).

### 2.2. Test Framework Fabrication

For the tests, a total number of 20 frameworks were fabricated. Five CAD-cast frameworks were produced using the same CAD design and materials. Another five frameworks were 3D printed from Cr-Co alloy powder (10–40 mm; Concept Laser) (Remanium Star CL; Dentsply Sirona, NC, USA) using selective laser melting (SLM) on a Concept Laser system (GE Additive, Cincinnati, OH, USA). To reduce internal stress, the frameworks were subjected to heat treatment at 1500 °C for one hour, followed by gradual cooling.

**Figure 2:**
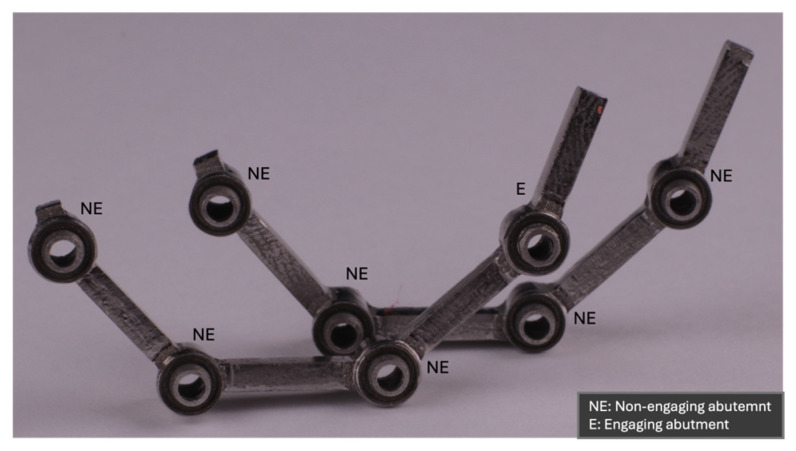
The difference between the two framework designs, which differ in their terminal abutment. The modified CAD framework exhibits an engaging terminal abutment, whereas other frameworks had only non-engaging abutments.

A modified CAD design featuring an engaging terminal abutment was used to produce five CAD-cast and five 3D-printed frameworks, following identical protocols (Figure 2). Each framework was mounted to the model with new implant screws (048.350; Straumann), torqued to 35 Ncm in the sequence 2, 3, 4, 1 using a digital torque meter (BTGE-G; Tohnichi, Tokyo, Japan). A custom-designed silicon stand ensured consistent framework positioning for gap measurement.

**Figure 3:**
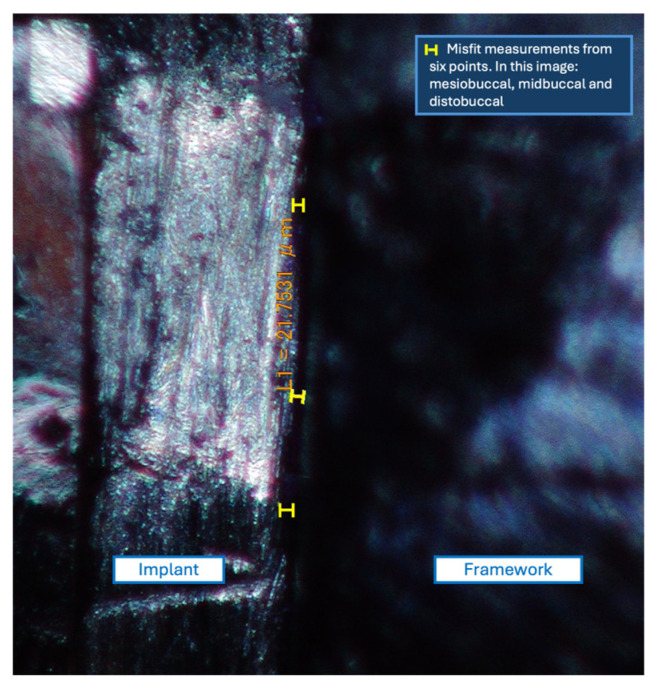
Misfit measurement using a scanning electron microscope. The gap between the implants and framework was measured at six points (mesiobuccal, midbuccal, distobuccal, mesiolingual, midlingual, and distolingual). This image shows the measurement points from the buccal side.

All specimens were cleaned in an ultrasonic bath with acetone and air-dried. Misfit was assessed via scanning electron microscopy (SEM) (FEI Apreo FEG SEM; Thermo Fisher Scientific) at 150× magnification. Measurements were taken from six reference points (mesiobuccal, midbuccal, distobuccal, mesiolingual, midlingual, and distolingual) marked with a fine-tip permanent marker (Sharpie; Newell brand, Atlanta, GA, USA) (Figure 3). The measurement procedure included calibration and validation steps to ensure accuracy and repeatability. To ensure reliability, measurement reproducibility was evaluated by performing repeated measurements on multiple specimens at defined reference points. These procedures aimed to confirm consistency and minimize measurement uncertainty in the misfit analysis.

### 2.3. Strain Gauge Placement and Calibration

**Figure 4:**
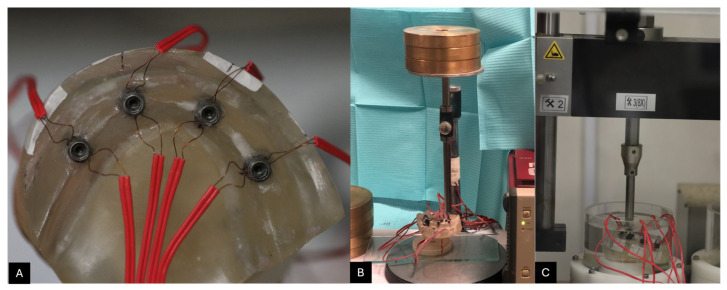
(**A**) Strain gauge installed on buccal and lingual sides of each implant. (**B**) Strain gauge calibration. (**C**) Positioning of the model in the cyclic loading chewing simulator (CS-4) with a 30-degree angle.

To further evaluate strain distribution, eight high-precision strain gauges (EA-06-015 and EH 120; Micro-Measurements Group, Romulus, MI, USA) were attached to the buccal and lingual sides of each implant (Figure 4A). The precise orientation of each implant’s long axis was determined using a digital surveyor (NDI; Bloomfield, CT), ensuring accurate placement of the strain gauges. Each gauge was positioned parallel to the implant’s axis with a 180-degree separation in the horizontal plane. To enhance adhesion, the implant surface was roughened using an extra-fine grain sandpaper disk (E.C. Moore CO, Inc., Dearborn, MI, USA) mounted on a straight handpiece (EXPERTmatic E10C; KavoKerr, Orange, CA, USA). The surface was subsequently cleansed using acetone-soaked gauze (Dukal Gauze Pads; Dukal Corporation, Ronkonkoma, NY, USA) to remove any residual particles.

The strain gauges (SGs) were attached using rapid-cure strain gauge adhesive (SG401; Omega Engineering, Norwalk, CA, USA) with gentle pressure using a piece of Teflon film (Omega Engineering, Norwalk, CA, USA) for 60 s, according to the manufacturer’s instructions (Figure 4). Each gauge was wired into a quarter-bridge circuit connected to a 10-channel strain monitoring system (Portable 10-Channel Strain Gauge Monitor; Omega Engineering, Norwalk, CA, USA). The output signals were digitized via an analog-to-digital converter (ADC Converter; Texas Instruments, Dallas, TX, USA) and recorded on a computer system (Figure 4B,C).

### 2.4. Preload Measurements and Cyclic Loading

A total of 100 screws (048.350; Straumann) were used on all frameworks; 80 screws were distributed to CAD-cast and three-dimensional printing (3DP) frameworks, and 20 screws were alternatively used on the PF framework to represent five running cycles of cyclic loading. The sample size was determined based on power analysis (0.91) using the G*Power sample size calculator software (version 3.1; University of Kiel, Kiel, Germany).

An indentation on the cantilever extension was marked 9 mm from the center of implant No. 4 to standardize the point of loading on the framework. For SG calibration, the master model was stabilized on the dental surveyor base using clear heat-cured acrylic resin (Clear Orthodontic Resin; Dentsply Sirona, Charlotte, CA, USA). A specially made load carrier plate, comprising a round metal plate fixed on a pointed-end metal rod, was attached to the dental surveyor.

A non-engaging abutment (048.605; Straumann) was tightened to implant No. 1 after removing the castable plastic part, and a new screw (048.350; Straumann) was used for each implant. The pointed end of the load carrier was placed on the tightened abutment, and the channels of the strain meter were zeroed.

A known load was incrementally applied up to 24 kg, increasing by 1 kg at a time, and the readings were recorded from the strain meter for SGs 1 and 2 in microstrain (µε). The calibration process was repeated three times for each implant, and the mean was used in the calculations. The same process was repeated for the other three implants. These repeated calibrations showed consistent results, indicating reliable measurement reproducibility.

For the experimental protocol, all SG channels were zeroed, and then each framework with a new set of screws was placed and torqued to 35 Ncm for implant Nos. 2, 3, 4, and 1 using a digital torque meter (BTGE-G; Tohnichi). Retorquing was performed after 10 min using the same sequence as the initial torque application [27,28,29]. The microstrain-based preload values were recorded prior to cyclic loading from all eight channels attached to the strain gauges (SGs), and the aggregate preload across all implants was calculated to determine the total preload for each framework. Measurement uncertainties arising from the calibration process were considered during data analysis to ensure accuracy in preload values.

**Figure 5:**
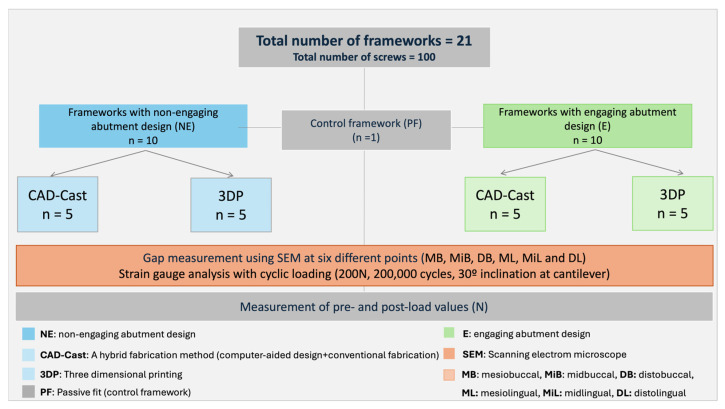
Workflow showing the different study groups and investigations.

The frameworks were then subjected to cyclic loading via a mechanical chewing simulator (CS-4; Mechatronik GmbH, Pleidelsheim, Germany). Each framework underwent cyclic loading with a 200 N force applied over 200,000 cycles, with a 30-degree inclination at the cantilever extension, positioned 9 mm from the center of the terminal implant in compliance with the International Organization for Standardization (ISO 14801:2017) [27,30,31]. Upon completing the cyclic loading phase, the post-cyclic preload values were documented after a 31-h interval. The recorded SG readings were subsequently converted into preload values (N) for each implant. A detailed workflow of the different investigated groups is shown in Figure 5.

### 2.5. Statistical Analysis

Statistical analysis was performed using SPSS software (Version 29.0.2.0, IBM Corp., Armonk, NY, USA). Normality of the data was assessed prior to analysis. A one-way analysis of variance (ANOVA) was used to compare mean gap measurements and preload values across different framework manufacturing techniques. Where significant differences were found, the Games–Howell post hoc test was applied for pairwise comparisons due to unequal variances among groups. Independent samples *t*-tests were conducted to compare engaging versus non-engaging abutment designs within each manufacturing group for both misfit and strain gauge readings. Pearson correlation analysis was performed to assess the relationship between implant–abutment misfit and abutment screw preload under cyclic loading. A significance level of α = 0.05 was used for all statistical tests.

## 3. Results

Gap measurements for each framework type were taken from six reference points (mesiobuccal, midbuccal, distobuccal, mesiolingual, midlingual, and distolingual) on each implant, with a total of 24 readings for each framework. The mean values were obtained from the readings of all frameworks per group and summarized in Table 1. The control framework (PF) exhibited the lowest mean gap (16.04 ± 2.6 μm), while the highest mean gaps were observed in the CAD-cast E (32.98 ± 1.43 μm) and 3DP E (32.96 ± 1.67 μm) frameworks. The CAD-cast NE and 3DP NE frameworks showed intermediate values of 29.2 ± 3.1 μm and 24.5 ± 1.05 μm, respectively.

**Figure 6:**
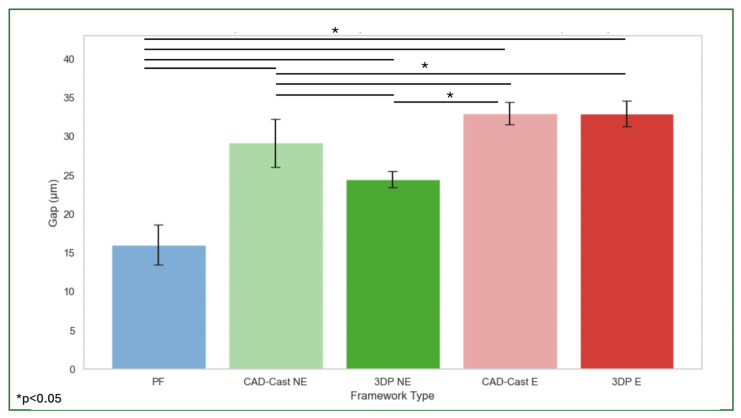
Gap measurement in μm within each group of manufacturing and abutment connection design. The one-way ANOVA revealed statistically significant differences in gap measurements across the five framework types (*p* < 0.05), except between CAD-cast_E and 3DP_E. Lines and stars represent statistically significant differences.

A one-way ANOVA revealed statistically significant differences in gap measurements across the five framework types (*p* < 0.05), except between CAD-cast_E and 3DP_E, which did not differ significantly. Post hoc comparisons using the Games–Howell test confirmed that the PF framework differed significantly from both engaging abutment groups (mean difference: 16.9 μm), whereas the difference between CAD-cast_E and 3DP_E was minimal (mean difference: 0.02 μm) (Figure 6).

An independent samples *t*-test comparing misfit between engaging (E) and non-engaging (NE) abutment designs showed significant differences in both CAD-cast and 3DP frameworks (*p* < 0.05). In contrast, no significant differences in strain gauge (SG) readings were detected between E and NE designs in either manufacturing group (*p* > 0.05) (Table 2).

Regarding preload, mean values for PF, CAD-cast, and 3DP frameworks were 173.4 N, 293.2 N, and 330 N, respectively. One-way ANOVA indicated no significant differences in preload values among frameworks under cyclic loading (*p* > 0.05). SG readings showed preload values of 293.2 N (NE) and 140.8 N (E) in CAD-cast frameworks and 330 N (NE) and 360 N (E) in 3DP frameworks (Table 3). Due to the large standard deviations, particularly in the CAD-cast and 3DP groups, a post hoc power analysis was performed. The estimated effect size (Cohen’s *f*) was approximately 0.25, and the achieved statistical power was approximately 60–65% for the given sample sizes. A Pearson correlation analysis indicated a weak positive but non-significant correlation between implant–abutment misfit and screw preload (r = 0.2, *p* = 0.4).

## 4. Discussion

Implant–abutment misfit might introduce uncontrolled strains that are exacerbated by occlusal forces and cause screw loosening and loss of preload [32,33]. Studies have shown that some level of misfit is inevitable in both ISFDPs and ICFDPs [13,33,34,35,36,37]. According to the results of this study, the first part of the null hypothesis can be rejected, as it showed that the CAD-cast manufacturing technique had a higher implant–abutment misfit than 3DP and PF, regardless of abutment design. This can be explained by human errors introduced during the casting procedures. The results are consistent with a study by Presotto et al., who compared ISFDPs fabricated using conventional, milled, and 3DP manufacturing methods and found that 3DP showed better implant–abutment connection misfit than casting or milling [11]. Here, it is noteworthy to mention that the CAD-cast NE framework used in the current study was selected as the control, although it is not considered a clinically fabricated gold standard such as a milled titanium framework. However, due to its widespread use in laboratory and clinical practice, this method offers a reproducible and standardized workflow that reflects conventional fabrication techniques still commonly applied in full-arch restorations. It thus may serve as a reliable baseline for comparing newer digital manufacturing methods under controlled in vitro conditions.

A systematic review by Bae et al. concluded that ISFDPs manufactured by 3DP had improved implant–abutment connection misfit values compared to ISFDPs manufactured by casting methods [17]. Interestingly, in the current study, frameworks containing a single E abutment exhibited higher misfit compared to those with all NE abutments. This may be due to the localized anti-rotational constraint introduced by the E abutment, which can hinder passive fit when not used symmetrically. The mechanical interference caused by a single point of engagement may lead to uneven seating of the prosthesis. Although the misfit was higher with the E abutment, it was still within the reported average micro-gap of the implant–abutment connection, which ranges from 1 to 49 µm [38]. The misfit level in the engaging abutment was also lower than the clinically acceptable limit of 150 μm, as reported in the literature, which validates the use of one E abutment design [39,40,41].

The second part of this study aimed to evaluate the effect of digital manufacturing technique and implant–abutment connection design on the screw preload of ICFDPs under cyclic loading. According to the results of this study, the null hypothesis stating that there are no differences in the amount of preload on the implant–abutment connection screw of different digital ICFDPs with different abutment designs upon cyclic loading cannot be rejected. This suggests that using frameworks with different manufacturing techniques and connection designs does not affect the preload of the implant–abutment connection screw under cyclic loading.

According to the results of this study, preload values differed numerically among the groups—most notably between the PF (173.4 ± 79.5 Ncm) and 3DP (330 ± 253.2 Ncm) frameworks—though these differences were not statistically significant (*p* = 0.5). The substantial standard deviations observed suggest high intra-group variability. A post hoc power analysis indicated an estimated effect size (Cohen’s *f*) of approximately 0.25 and a statistical power of around 60–65%. These findings suggest that the lack of significance may be due in part to insufficient power to detect medium effects, highlighting the influence of variability and sample size limitations on the statistical outcome. Interestingly, while the CAD-cast group showed higher preload with non-engaging abutments, the 3DP group demonstrated the opposite trend, with engaging abutments showing higher preload values. This discrepancy may be attributed to differences in surface characteristics and material behavior inherent to the manufacturing methods. The 3DP frameworks, often fabricated via selective laser melting, typically exhibit a rougher surface texture compared to the smoother cast frameworks, which could increase friction at the implant–abutment interface and thus affect the tightening dynamics and preload. Additionally, the insertion force variability during screw tightening may differ between the more rigid CAD-cast frameworks and the potentially more elastic or dimensionally variable 3DP frameworks, influencing the clamping force achieved. Material properties such as modulus of elasticity and microstructural differences might also contribute to how deformation under load affects preload values. These factors, combined with prosthetic misfit, may explain the observed preload behavior. Previous studies have shown that greater misfit can increase preload due to deformation-induced forces during tightening [42,43]. Hegde et al. evaluated the SGs response to a known level of misfit in ISFDP. They found that the preload level significantly increased with increasing levels of misfit [43]. Clinically, all preload values in this study fell within or near the accepted range of 200–400 N considered sufficient for abutment screw stability and resistance to occlusal forces. Despite the lack of statistical significance, these findings highlight the complex interplay between manufacturing technique, abutment design, and biomechanical behavior. Further research with larger sample sizes and detailed surface analyses is recommended to elucidate these relationships and their implications for clinical performance.

Epprecht et al. compared preload at the implant–abutment connection level in NE and E abutments of four-unit zirconia after torque application. They found that the implant–abutment connection design had no significant effect on the preload level of the implant–abutment connection screw [2]. This is in accordance with the results of this study, which showed that regardless of the manufacturing technique, implant–abutment connection design had no significant effect on the screw preload. One possible explanation for this similarity is that both studies used multi-unit prosthetic designs, where the mechanical influence of a single engaging abutment may be minimized by the overall splinting effect of the framework. Additionally, the preload measurements in both studies were taken after standardized torque application, which may have overshadowed subtle differences in connection design. It is also possible that the precision of digital manufacturing methods reduces the variability introduced by abutment geometry, resulting in more consistent preload outcomes across designs. Another factor was suggested by Fan X et al., who showed a positive correlation between ISFDPs with cantilever design and resistance to screw loosening [44]. This suggests that cantilever configuration may play a role in mechanical stress distribution and preload maintenance. However, it is important to acknowledge that some findings in the literature appear to contradict the interpretations presented here, particularly regarding the relationship between preload and prosthetic misfit. For instance, Hegde et al. [43] reported a significant increase in preload with increasing misfit, suggesting that deformation at the implant–abutment interface during tightening can enhance the clamping force. Conversely, Fan et al. [44] observed that frameworks with lower misfit exhibited higher preload values, which challenges this assumption. One potential explanation for these inconsistencies lies in methodological differences, particularly in strain gauge (SG) placement. While our study positioned SGs at the abutment screw level to directly measure preload, other studies placed SGs on the framework or superstructure, potentially reflecting strain patterns not solely attributable to screw tension. These variations in experimental setup may influence preload readings and complicate direct comparisons. Thus, the relationship between misfit, preload, cantilever design, and screw stability remains complex and likely multifactorial. Future studies should explore how different cantilever lengths or configurations influence preload and resistance to screw loosening, especially in the context of various abutment and manufacturing designs.

In CAD-cast frameworks, higher preload was observed in NE abutments, which is in accordance with a previous study [2]. This can be attributed to the improved fit in the NE abutment compared to the E abutment, which requires less deformation to close the gap during loading. However, in 3DP frameworks, data showed higher preload levels in E abutments, which could be explained by the limited path of insertion in frameworks with one E abutment compared to those with all NE abutments; it can also be attributed to the roughness of the 3DP framework connection and the need for additional surface characterization, which might affect the material’s characteristics [45]. It can be argued that modifying the location and number of the E abutments might produce different results. Furthermore, the loading position was restricted to the cantilever extension. Applying an equal force distribution could yield different results. Further investigations should compare screw preload of ICFDPs with different E abutment locations and numbers and under various occlusal conditions.

In the current study, selective laser melting (SLM) was employed for the fabrication of 3D-printed metal frameworks due to its high accuracy and mechanical properties, which are particularly essential for full-arch implant-supported prostheses [46,47]. However, it is important to note that other additive manufacturing technologies—such as Fused Deposition Modeling (FDM) and photopolymer-based methods like Stereolithography (SLA) and Digital Light Processing (DLP)—have also been explored in dental research [48]. Although these techniques are primarily used for prototyping or fabricating resin-based components, some studies have investigated their potential in producing dental prostheses and even implant components [49]. However, further research is warranted to assess their viability for definitive implant-supported prostheses.

One important limitation of the current study is the chewing simulation. Although cyclic loading with a 200 N force applied at a 30-degree inclination simulates relevant occlusal forces based on ISO 14801:2017 standards [50], it does not fully replicate the complex, multidirectional, and distributed nature of chewing forces encountered clinically. Real masticatory loads vary in direction, magnitude, and frequency across multiple contact points. Future studies should incorporate multi-point loading and dynamic occlusal force simulations to better mimic in vivo conditions and more accurately assess the biomechanical behavior of implant-supported prostheses.

Another limitation is that the tested frameworks with only one engaging (E) abutment per specimen limit the understanding of how multiple engaging abutments might influence preload behavior and overall prosthesis fit. The effect of varying the number and distribution of engaging versus non-engaging abutments remains unclear. Future studies should explore different E/NE configurations to better simulate clinical scenarios and improve the generalizability of the findings.

## 5. Conclusions

Within the limitations of this study, the following can be concluded:

⚬ 3DP technique produces prostheses with better implant–abutment fit than the CAD-cast.
⚬ Using a single engaging terminal abutment results in an implant–abutment misfit that remains within acceptable limits.
⚬ Different manufacturing techniques produce approximate preload values at the implant–abutment connection screw when the prosthesis is loaded.
⚬ Using only non-engaging abutments results in higher preload values with the CAD-cast manufacturing technique.
⚬ Using a single engaging terminal abutment results in higher preload values with the 3DP manufacturing technique.

## Figures and Tables

**Table 1:** Gap measurements (μm) at buccal and lingual points for each framework, with mean and standard deviations.

	PF		CAD-cast_NE		3DP_NE		CAD-cast_E		3DP_E	
	Mean (BP)	Mean (LP)	Mean (BP)	Mean (LP)	Mean (BP)	Mean (LP)	Mean (BP)	Mean (LP)	Mean (BP)	Mean (LP)
Implant No. 1	12.00	16.33	26.10	25.70	24.67	24.57	30.53	34.53	34.00	35.33
Implant No. 2	12.00	16.33	30.50	28.90	24.00	24.00	33.93	33.73	32.00	31.33
Implant No. 3	17.33	17.33	32.80	31.60	22.67	22.67	31.93	32.07	32.00	31.67
Implant No. 4	18.00	19.00	27.40	30.80	24.00	24.00	34.53	32.60	35.33	32.00
Mean ± SD	16.04 ± 2.60		29.20 ± 3.10		24.05 ± 1.05		32.98 ± 1.43		32.96 ± 1.67	

PF: passive fit; CAD-cast: a combined computer-aided design framework and a conventional fabrication method; 3DP: three-dimensional printing; NE: non-engaging; E: engaging; BP: buccal points; LP: lingual points.

**Table 2:** Independent sample *t*-test comparing the effect of implant–abutment connection design on SG readings of CAD-cast and 3DP frameworks.

Framework Manufacturing Technique	Abutment Connection Design	Mean ± SD (N)	F	Df	Mean Difference	Sig
CAD-cast	NE	293.2 ± 266.5	2.2	8	152.4	0.25
CAD-cast	E	140.8 ± 87.4	2.2	8	152.4	0.25
3DP	NE	330 ± 25	46	8	30	0.79
3DP	E	360 ± 25	46	8	30	0.79

**Table 3:** One-way ANOVA of the effect of framework manufacturing technique on abutment screw preload in non-engaging abutment design.

Framework Manufacturing Technique	Mean ± SD (Preload in Ncm)	*p*
PF	173.4 ± 79.5	0.5
CAD-cast	293.2 ± 266.5	0.5
3DP	330 ± 253.2	0.5

## Data Availability

The original contributions presented in this study are included in the article. Further inquiries can be directed to the corresponding author.

## Abbreviations

The following abbreviations are used in this manuscript:

CAD	Computer-aided design
CAD/CAM	Computer-aided design/computer-aided manufacturing
E	Engaging abutment
ICFDP	Implant-supported cantilever fixed dental prostheses
NE	Non-engaging abutment
PF	Passive fit
SEM	Scanning electron microscope
3DP	Three-dimensional printing
